# Insights from modelling malaria vaccines for policy decisions: the focus on RTS,S

**DOI:** 10.1186/s12936-021-03973-y

**Published:** 2021-11-18

**Authors:** Katya Galactionova, Thomas A. Smith, Melissa A. Penny

**Affiliations:** 1 Swiss Tropical and Public Health Institute, 4051 Basel, Switzerland; 2 University of Basel, 4001 Basel, Switzerland; 3 European Center of Pharmaceutical Medicine, Brombacherstrasse 5, 4057 Basel, Switzerland

**Keywords:** Malaria vaccines, Modelling and simulation, Economic evaluation, Malaria control, Product development

## Abstract

Mathematical models are increasingly used to inform decisions throughout product development pathways from pre-clinical studies to country implementation of novel health interventions. This review illustrates the utility of simulation approaches by reviewing the literature on malaria vaccine modelling, with a focus on its link to the development of policy guidance for the first licensed product, RTS,S/AS01. The main contributions of modelling studies have been in inferring the mechanism of action and efficacy profile of RTS,S; to predicting the public health impact; and economic modelling mainly comprising cost-effectiveness analysis. The value of both product-specific and generic modelling of vaccines is highlighted.

**Supplementary Information:**

The online version contains supplementary material available at 10.1186/s12936-021-03973-y.

## Background

The long history of innovation in malaria vaccines has demonstrated varied success [1–3]. July 2015 marked a significant breakthrough with licensure by European regulators of the RTS,S recombinant protein-based vaccine, after two decades of testing in clinical trials [4]. RTS,S/AS01 demonstrated sustained protection against *Plasmodium falciparum* in an extensive phase 3 evaluation in 11 trial sites in Burkina Faso, Ghana, Kenya, Tanzania, Malawi, and Mozambique [5]. Efficacy against clinical disease was initially high but waned quickly, averaging 36.3% in children vaccinated at 5–17 months of age (95% Confidence Interval (CI) 31.8–40.5%) in a 4-dose schedule with at least 32 months follow-up, and 25.9% in those 6–12 weeks old (95% CI 19.9–31.5%) [6].

Following formal review of both trial data and modelling evidence, in 2016 the World Health Organization (WHO) recommended evaluation via a large-scale pilot implementation, to resolve safety signals identified in phase 3, to generate evidence of mortality impact, and to demonstrate feasibility of a four-dose vaccination schedule via routine Expanded Programme of Immunization (EPI) schedules in endemic Africa [7]. The Malaria Vaccine Implementation Programme (MVIP) [8] is currently on-going in Ghana, Kenya, and Malawi. At the time of writing in 2021, a joint WHO Malaria Policy Advisory Group (MPAG) and WHO Strategic Advisory Group of Experts on Immunization (SAGE) recommendation was made on widespread use of the RTS,S/AS01 malaria vaccine among children in sub-Saharan Africa and in other regions with moderate to high *P. falciparum* malaria transmission. The recommendation is based on results from the first 24 months safey and efficacy evidence from the MVIP alongside efficacy outcomes from a separate clinical trial on RTS,S use as a seasonally targetted intevention with or without seasonal malaria chemoprevention [9].

RTS,S clinical development has been accompanied by mathematical modelling with questions ranging from the mechanism of action to the potential macroeconomic impact. This agenda adapted over time to new knowledge, regulatory developments, changes in the burden of malaria, and to different objectives and aspirations of funders and intervention programmes. The objectives, methodologies and key findings of this modelling are reviewed here, beginning with a historical overview. A summary table highlighting findings of selected key papers (Additional file 1: Table S1) is provided as a resource for analysts and policy-makers.

## History of modelling of malaria vaccines

Modelling of malaria with mathematical representations of transmission dynamics began with Ross’ deterministic population model [10], extended in the 1950s by Macdonald to consider malaria in mosquitoes and used as the theoretical rationale for elimination programmes based on indoor residual spraying, then the most effective available means of reducing transmission [11]. These models did not consider effects of acquired immunity on malaria in humans. Many extensions of the Ross-Macdonald model have been developed, including in the late twentieth century the incorporation of immunity and parameterization with field data [12] to the limited extent then feasible. When the first clinical studies were demonstrating the potential of recombinant sporozoite vaccines [13] these compartment models were extended to include vaccination [14, 15], showing that at high coverage stage-specific malaria vaccines can induce complex transmission dynamics even in relatively simple models [16]. Such deterministic population models have continued to be used to analyse the principles of transmission reduction by vaccines [17] and the threshold conditions for elimination [18], especially for settings with very low transmission, such as in South East Asia [19, 20] where there are fewer complicating effects of superinfection and acquired immunity.

From the 1980s until the renewed focus on elimination after the 2007 Gates Malaria Forum [21], most malaria programmes focused almost exclusively on reducing malaria morbidity and mortality, irrespective of transmission-reducing effects. RTS,S was thus evaluated for preventing paediatric clinical malaria, not as an elimination tool. The need to consider effects on morbidity and mortality motivated the development of stochastic individual-based models (IBMs), which can more readily be extended to capture detailed aspects of malaria outcomes, such as pathogenesis and heterogeneity of infection and immunity between individuals than can population models. OpenMalaria [22–25] was the first such model used explicitly for simulating RTS,S.

The original OpenMalaria model for EPI vaccination [26] used estimates of efficacy in infants from the initial RTS,S phase 2 trial [27]. Because the vaccinated group were only a small part of the population, the impact on population levels of transmission was estimated to be minimal, making the detailed model for infectiousness of the human host [28] unnecessary for modelling impacts of EPI vaccination. The vaccination effects in this microsimulation could be emulated by a simpler compartment model [19, 29], but the more complicated model remained necessary to show that this was the case. The same logic applied to many other aspects of malaria epidemiology, which have been included in IBMs presumptively in case of important effects on outcomes. The availability of high-speed computing made the additional computational overhead of IBMs less important, and the Imperial College model [30] and EMOD DTK [31] have been widely used for vaccine modelling in addition to OpenMalaria. Once the phase 3 data were available, these three dynamic models were employed alongside a static model developed by GSK [32] in a consensus exercise aligned with WHO processes [33] to support WHO guidance on RTS,S deployment [34]. Additional file 1: Table S1 documents methodologies and assumptions of these models about malaria pathogenesis and immunity, relating these to differences in the predictions. These models have continued to be used to address questions about future roles of RTS,S during the current phase of pilot deployment.

## Models for mechanism of action and efficacy profile of RTS,S

Models of PEVs are parameterized via effects on the transmission rate from mosquitoes to humans. Simple reductions in this parameter can be assumed if the objective is only to clarify the principles of vaccine-induced dynamics. In quantifying the impact of a real-world vaccine, estimating this reduction can be challenging because most trials focus on short-term effects on clinical incidence and only indirectly estimate the immediate effect in reducing force of infection. Simulations of hypothetical vaccine profiles show that both average efficacy and the time-course of its decay are important for long-term impact [26, 30, 31, 35]. Prior to availability of long-term follow-up data this was addressed only by sensitivity analyses (which rarely considered decay as rapid as that subsequently measured).

In principle, efficacy and its decay rate might vary by characteristics like age, previous exposure, and sex, making desirable a surrogate measure of efficacy that could be used in models of impact. The effects of RTS,S/AS01 on the rate of acquisition of new blood stage infections, on the initial inoculum of blood stage infection, and on the multiplicity of infections are all hypothesized to result from the induction of anti-circumsporozoite (CS) antibodies and/or CD4 + T cells [36, 37]. A model of antibody dynamics fitted to longitudinal data on CS antibody titres from the phase 3 trial, suggested using CS antibody titres as a surrogate for protection against infection [38] and hence for other downstream effects. The pattern of waning efficacy mirroring the rapid waning of anti-CS antibody titres in the first six months followed by slower waning over the next four years, while the boosting effect of the fourth dose was limited, supporting the use of antibody titres as a surrogate. The efficacy profile in the Imperial College IBM [39–41] was informed by this analysis of antibody titres. A subsequent within-host model, fitted to data from the challenge trial of fractional dosing, has found that IgG avidity can explain even more variation in vaccine efficacy than can titre alone [42].

Once available, aggregated 3-monthly phase 3 trial incidence data were used to infer efficacy profiles for OpenMalaria, EMOD GTK and the GSK model. These profiles comprised estimates of efficacy against infection, its degree of homogeneity, and its decay over time [34]. There was a high initial post third dose efficacy against infection (> 75%) in the 5–17 month cohort, with fast waning during the first 12 months post third dose [34]. By fitting different functions to the decay of effect over time, protection was estimated to last up to 7.5 years in two models and over 10 years in the other two. The boosting effect of the fourth dose was estimated be about half of the primary series with slower waning compared to the third dose [43]. There were discrepancies between models in estimated vaccine profiles, reflecting differences in model structure and in case definitions, underlying relationship between the parasite prevalence and clinical incidence, assumptions on immunity and differences in other calibration datasets [34]. Predictions of the efficacy decay shape following the fourth dose were more uncertain due to the short follow-up (the fourth dose was administered 18 months after the third (14 months follow-up)) and the reduction in statistical power in the trial (half of the vaccinated cohort received the fourth dose).

## Individual-based models of the impact of malaria vaccines

### Modelling based on hypothetical efficacy profiles

Before RTS,S phase 3 trial data were available, PEVs with various hypothetical profiles and deployment modalities were simulated, not only through the EPI [24, 30, 35, 44, 45], alone or in combination with other interventions [30, 31]; and on exploring the minimum efficacy profiles needed to reach an explicit prevalence reduction or elimination target [30, 31, 35]. The different simulation models were calibrated with different field data, monitor different clinical outcomes, and vary in case definitions, the extent of spatial heterogeneity and interactions captured, in treatment of multiplicity of infection, assumptions on immunity, and structural elements underlying the biological phenomena (Additional file 1: Table S1). Nevertheless, they make many similar predictions, for instance all agreeing that unless there are additional interventions, vaccination programmes resulting in rapid decreases in clinical disease, but not elimination, will lead to increases in clinical disease many years later as population-level immunity is lost [17, 26, 45]. Sensitivity analysis demonstrated the importance of the transmission intensity and the pattern of decay of vaccine efficacy in determining impact. Some uncertainties though, for instance those relating to the potential evolution of insensitivity to vaccines [46, 47], fall outside the scope of such analysis and remain unaddressed.

Modelling of hypothetical vaccine profiles led to an appreciation of the relative importance of vaccine properties for different operational targets, be it interruption of transmission or burden reduction. This revealed that duration of protection was more important than initial efficacy for burden reduction [24, 26, 35], with initial efficacy more critical for interruption of transmission [24]. The distribution of vaccine impact in the population depends on the vaccine half-life: the shorter the half-life, the more impact concentrates in younger individuals [48]. A vaccine (like RTS,S) providing partial protection to more people is preferable to one providing complete protection to some and no protection to others [26, 35], giving greater population impact and less age-shifting of clinical disease.

Different modelling studies broadly agreed on the potential of paediatric malaria vaccines to significantly reduce *P. falciparum* morbidity and mortality in young children across a range of vaccine efficacy profiles and transmission intensities [24, 26, 31, 44]. These studies also agreed that the narrow target age-range means that partially efficacious vaccines deployed through the EPI would have minimal impact on overall transmission or prevalence [24, 26, 31, 44].

In higher transmission settings modelled vaccine effectiveness is lower [24, 26, 49, 50] and vaccine programmes are expected to induce a shift in morbidity to older ages, which sets in sooner the higher the transmission [50]. These effects are offset by the higher burden at high transmission so that, except at the highest transmission levels, there is an increase in impact with transmission [24, 26, 49]. Effectiveness is also higher where transmission is more homogeneous [24]. Malaria vaccines would consequently have the most impact in moderate to high transmission settings with saturated coverage of existing tools [30, 31, 45] and where outdoor biting vectors limit the effects of vector-control [31, 45].

### Modelling based on the RTS,S/AS01 Phase 3 efficacy profile

Once the RTS,S/AS01 efficacy profile was estimated from phase 3 data, simulation results could be translated into precise projections of public health impact. At values of prevalence in children 2–10 years old between 10 and 65% with high coverage of LLINs (68%) and moderate treatment coverage (45%) the vaccine deployed in the four-dose schedule in children aged 5 to 17 months was estimated to avert a median of 116,480 (range across model predictions, 31,450–160,410) clinical cases and 484 (189–859) deaths per 100,000 fully-vaccinated children (FVC) over 15 years [34].

Modelling within the consensus exercise to support WHO guidance on RTS,S deployment highlighted two conclusions that differed from naïve interpretations of phase 3 results. Firstly, all models allowed for health care system performance in inferring likely mortality impact under implementation conditions and predicted a net beneficial impact on severe disease and mortality with the primary schedule [34]. This contrasted with the trials because most cases in the trial were promptly and effectively managed [34] leading to low placebo mortality, unlike in routine care in the resource-poor settings for which the vaccine is intended. The currently on-going pilot evaluation of the vaccine aims to collect relevant severe disease and mortality data complementary to these model inferences, that should feed into the discussion of inferences about mortality in formulating global guidance.

The second apparent discrepancy was that the trial data were interpreted as suggesting that the fourth dose was necessary for protection against severe outcomes [51] while all models predicted impact on severe outcomes in both three and four dose schedules. Two groups reporting only minor incremental benefit of the fourth dose [34]. A three-dose schedule improves the operational feasibility of the vaccine implementation since all three doses will usually be delivered along with other routine visits or at most requiring one additional visit outside of the routine schedule. Moreover, simulations of public health impact of vaccines with modified profiles suggest an advantage in prioritizing initial efficacy over duration [48]. This all implies that should the effectiveness of the vaccine against severe outcomes with the primary series be established in the pilot evaluation, a three-dose schedule may be preferable.

Further modelling translated the efficacy profile into predictions of the impact of RTS,S programmes in each endemic African country [39, 49] using geographically-specific estimates of malaria transmission potential derived by the Malaria Atlas Project [52]. The considerable reduction in transmission in the last two decades [52] meant that by 2015 a large proportion of the population of Africa were exposed at levels where the models all predicted a substantial public health impact, despite the moderate efficacy and rapid waning of effect. The significance of these outcomes and congruence of the models supported further investigation of RTS,S.

Additional modelling has also considered alternatives to the use of RTS,S only as a morbidity control tool in young children. Mass vaccination with RTS,S could have synergistic effects with mass-drug administration because the combined effect can reduce onward transmission below critical thresholds, leading to dramatic prevalence reductions and in few settings, transmission interruption [53]. Synergistic effects with seasonal malaria chemoprevention (SMC), targeting specific vulnerable populations (such as forest workers with limited options for vector control) and use in outbreaks have also been explored [54].

## Modelling the economics of malaria vaccines

Malaria vaccine programmes are likely to have complex economic impacts, but there has been little modelling of their macroeconomic effects, an exception being the analysis by Yerushalmi et al. using a general equilibrium model of the economy of Ghana [55]. Most of the literature consists of cost-effectiveness analyses (CEA) and budget impact analyses (BIA) from the provider or donor perspectives, with the key question for decision analysis from the perspective of a country policy maker being whether or not to introduce RTS,S as an additional malaria control measure in a setting where other vector control and curative interventions are already deployed at scale [33].

### Costing of vaccination

The initial OpenMalaria analyses included prospective costing studies of vaccine implementation within the EPI [56] and other delivery channels [57] in Tanzania. Costing of vaccine implementation was subsequently expanded to six high burden countries by Galactionova et al. [58] also addressing uncertainty about the cost of vaccine delivery outside of the routine schedule. Further detailed costing has been carried out for the pilot countries [59] and Winskill et al. [40] extended this to 43 endemic countries, allowing for decreasing marginal returns to scale at high levels of coverage. Variations within and across countries are important and the unknown future price per dose and wastage rate for this candidate vaccine adds substantially to the uncertainty about the actual costs of implementation.

These analyses have consistently found that wastage and drop-off assumptions have substantial implications. Despite great variation in service delivery cost between countries, cost differences between the three- and the four-dose schedules are relatively small compared with the anticipated price of the vaccine (which remains a major source of uncertainty).

### Cost-effectiveness and budget impact analysis

CEA carried out for a range of settings and hypothetical efficacy profiles indicated likely ranges of Incremental Cost-Effectiveness Ratios (ICERs) for introducing RTS,S, while emphasizing that duration of protection and vaccine price are the main sources of uncertainty [57, 60, 61]. Studies carried out before the phase 3 trial established the efficacy profile, indicated that RTS,S will be most attractive in moderate to high transmission settings where transmission has already been reduced by existing tools [30, 31, 45] and where there is residual transmission due to outdoor biting vectors [31, 45]. In the absence of information on either vaccine price or duration of protection, sensitivity analyses established that these variables, alongside the transmission intensity are parameters with the largest impact on ICERs [32, 34, 35, 61, 62], alongside the parameters of the pathogenesis model and case fatality rates (which dominate the uncertainty in vaccine impact on mortality [61]). Uncertainty in mortality models is a major contributor to uncertainty in impact of any malaria intervention because there is little data on the frequency and outcome of severe malaria in the community [63]. It also drives uncertainty in estimated ICERs as over 90% of malaria disability-adjusted life years are accounted for by mortality.

The earlier publications established the basis for the CEA carried out within the 2016 consensus process. Informed by the phase 3 efficacy profile, this found a minimum in the ICERs between an EIR of 2 and 20 infectious bites per annum, confirming that RTS,S/AS01 would probably be good value-for-money in moderate to high transmission settings at high coverages of other preventive and curative interventions [34]. The ICERs for the four consensus models for EPI deployment at an assumed price of $5 per dose was estimated at $87 (range 48–244) per DALY averted in *Pf*PR_2-10_ between 10 and 65%, higher at *Pf*PR_2-10_ at or below 10%, broadly consistent with the values estimated before the phase 3 data were available. This corresponds to high cost-effectiveness based on conventional GDP per capita thresholds.

Subsequent studies have used country-specific data in CEAs of RTS,S for other specific countries including Malawi [64] and Bangladesh [65]. OpenMalaria provided estimates for all 43 countries of endemic Africa [66]. While consistent with predictions for generic prevalence profiles [34] this study gave overall higher ICERs with (median $136 per DALY, range 116–220) in countries with median population-weighted prevalence in children above 10%. The analysis allowed for within country heterogeneity in transmission and seasonality, national-level case-management coverage, costs of vaccine service delivery and treatment, and local efficiency of programmes (modelled using country DTP3 and MMR2 coverage proxies allowing for drop-off between doses in the primary series). Transmission is the main driver of impact and cost-effectiveness but there is substantial variation in estimated cost-effectiveness within the narrow transmission brackets because of these other factors. Similarly, the GSK model has been used for CEA for 41 countries, comparing EPI deployment with child vaccination (which appeared more cost effective in most countries) [67].

These CEAs address the issue of value-for-money for donors, but strategic planning at country level needs to consider prioritization of vaccination in the context of other possibilities for intervention, for instance using constrained optimization approaches to identify the role of RTS,S. The Imperial College team compared ICERs for vaccine scale-up with those of other malaria interventions [39, 40, 68]. Hypothetical scale-up trajectories were defined by considering at each possible 10-percentage point increment, the next most-cost effective intervention coverage combination, until all interventions reached coverage of 100%. This analysis suggested prioritizing LLINs, IRS, and SMC over RTS,S [40]. However, as coverage increases, effectiveness and cost of interventions become increasingly non-linear, depending on the underlying health systems infrastructure and the incremental resource needs to increase coverage beyond current capacity. A consequence is that the relative ranking of interventions is highly uncertain when coverages are saturated.

Sauboin et al. [69] improved on the framing of the question by explicitly evaluating scale-up of combinations of interventions in Ghana. They concluded that introducing RTS,S would be the optimal first step in scale-up from current coverages, for reducing under-five malaria mortality at the lowest cost, followed by SMC in relevant areas, and then by further scaling-up of IRS and LLINs. The contrasting conclusions of these two studies are largely driven by constraints imposed on the level of coverage that could be achieved with each intervention and the costing of inefficiencies, with Sauboin et al. [69] assuming constraints that are more favourable to RTS,S. For instance, LLIN coverage was allowed to scale-up only from a baseline of 54% to a maximum of 60%, while incremental impact of RTS,S was assessed with a step-size of 10%. The cost assumptions also favoured RTS,S by including the cost of unused LLINs (40% of nets distributed) and by implicitly assuming 100% coverage of the full four-dose vaccination schedule, while Winskill et al. [39] allowed for drop-off between doses.

There are other potential incongruities that are difficult to assess based on the information in the paper that could have further biased the evaluation undermining the effectiveness of vector control (i.e. it is not clear for instance how the translation from EIR reduction (enabled by IRS and LLINs) to clinical disease was implemented and whether individual protection from LLINs were captured (much smaller than transmission effects, but further biases down the effectiveness of LLINs compared to RTS,S) [70]. The prioritization suggested by this optimization framework might not be optimal with respect to the outcome of interest because it ignores treatment, which is the only intervention that directly impacts the probability that an infection proves fatal.

In interpreting all such analyses it is important to consider whether the analysis is conducted from a societal perspective, or that of a manufacturer, donor, or a national programme. Similarly, the unaddressed systems factors and other setting-specific constraints are rarely explicit.

## Conclusions

Mathematical models have roles throughout the development of novel health interventions. In silico evaluation of hypothetical tools and deployment regimes is an important tool supporting decisions on what to evaluate in trials. This is especially relevant to malaria vaccines, given the profusion of potential products, dosing regimens, transmission contexts, operational targets, and alternative interventions competing for resources. Vaccines cannot replace existing malaria preventive, diagnostic, and treatment measures [71] and early in vaccine development programmes modelling is needed to support alignment of use-cases gaps (such as underserved demographic groups) in the existing intervention portfolio, and to help identify synergies.

Despite broader potential value, most malaria vaccine modelling has so far been focussed on incrementally enhancing models of RTS,S, assuming pre-specified deployment regimens. Decision-making frameworks developed by PATH-MVI [72] and subsequently the WHO Malaria Vaccine Implementation Project (MVIP) were used to guide the evaluation and modelling efforts towards outcomes that were prioritized by key stakeholders, and to ensure continuity and transparency in policy development. Representation in these working groups placed modellers in direct dialogue with global stakeholders. The benefits of focusing on EPI vaccination with RTS,S included the encouragement of extensive use of field data to estimate efficacy profiles and parameters of IBMs, endowing projections of RTS,S impact with unusual precision. This focus also provided opportunities for the development of IBMs and workflows for the increasingly demanding computational methods required. The consensus exercise built around ensembles of models has been exemplary in providing input to guidance and policy development, helping manage expectations when it became evident that malaria vaccination is no silver bullet. Modelling platforms are now well-placed to apply state-of-the-art methodologies to estimating incremental benefits of novel deployment regimens (such as fractional dosing of RTS,S [48]), next generation vaccines, and other novel malaria tools, within the increasingly complex landscape of malaria programmes.

Reliable estimation of the RTS,S efficacy profile has been especially important for CEA and decision analysis establishing value-for-money of RTS,S, but implications for where RTS,S should be deployed are very sensitive to the framing of the decision problem. The contrasting conclusions of Sauboin et al. [69] and Winskill et al. [39] on the position of RTS,S within a control strategy exemplify this. Partly this is because careful consideration is needed of the health systems factors constraining scale-up and of the cost functions. Uncertainty about vaccine pricing remains an elephant in the room of economic modelling carried out in the public domain. Affordability will depend on whether vaccine is paid for by domestic funding, GFATM, or GAVI, and on vaccine pricing.

Modelling, economics, and health systems expertise are all needed in bodies framing and guiding health policy development, and the experience gained for vaccines is highly relevant to other partially efficacious new tools against malaria. The continued collaboration between modelling groups and WHO will continue to be key to ensuring these disciplines effectively support global recommendations on new health technologies.

## Supplementary Information

**Additional file 1: Table S1.** Overview of key malaria models that have supported policy guidance on RTS,S.

## Data Availability

Not applicable.

## Abbreviations

WHO: World Health Organization
PATH-MVI: Malaria vaccine initiative at PATH
GFATM: Global Fund against AIDS, Tuberculosis and Malaria
GAVI: GAVI—The vaccine alliance
EPI: Expanded Programme for Immunization
IBM: Individual-based model
DTP3: 3Rd dose of Diphtheria, Tetanus, Pertussis vaccine
HL: Half-life of the vaccine effect
VE: Vaccine Efficacy against force of infection from the time of completing the full vaccination schedule (initial efficacy)
FIC: Fully Immunized Child (a child that received all three doses of the vaccine)
CMYP: Country Multi-Year Plan for Immunization
SSA: Sub-Saharan Africa
ICER: Incremental cost-effectiveness ratio
