# Supplementary material for: Insights from modelling malaria vaccines for policy decisions: the focus on RTS,S
Source: Malar J. 2021 Nov 18;20:439. doi: 10.1186/s12936-021-03973-y (PMC8600337; doi:10.1186/s12936-021-03973-y)
Supplement: Supplementary file 1 — Additional file 1: Table S1. Overview of key malaria models that have supported policy guidance on RTS,S. [file 12936_2021_3973_MOESM1_ESM.pdf]

Supplementary Information to:

Insights from modelling malaria vaccines for policy decisions

Katya Galactionova[1,2,3], Thomas A. Smith[1,2], Melissa A. Penny[1,2]

1. Swiss Tropical and Public Health Institute, 4051, Basel, Switzerland.

2. University of Basel, 4001 Basel, Switzerland.

3. Current address: European Center of Pharmaceutical Medicine, Brombacherstrasse 5, 4057 Basel, Switzerland

Table S 1. Overview of key malaria models that have supported policy guidance on RTS,S

	GSK	Imperial College	EMOD DTK	OpenMalaria
Key representative publications	[1, 2]	[3-6]	[7-9]<br>[10-12]	[13-21]
Accessibility	A deterministic version of the model has been developed in MS Excel which can be shared upon request	Interface to published model allows the user to run the model on their PC. Incorporates estimated prevalence, vector species, seasonality, ITN coverage and treatment at ADMIN1 level that the user can change. https://www.imperial.ac.uk/malaria-modelling/tools-and-data/	Latest Windows release of EMOD malaria model and documentation can be downloaded from idmod.org/software	Code is open source. Runs on Windows, Linux and Mac. Full documentation is found at https://github.com/SwissTPH/openmalaria/wiki
Seasonality	No	Yes	Yes	Yes
Heterogeneity in exposure	Yes	Yes	Yes	Yes
Blood-stage parasite densities modelled	No	No	Yes	Yes
Parameterization for clinical incidence	Fitted to RTS,S phase 3 trial data[22]	Fitted to cross-sectional age-incidence data from 23 sites in Africa capturing differences between active and passive case detection[4]	Calibrations to 4 sites for parasitaemia (Nigeria, Tanzania), Dielmo and Ndiop (Senegal)[23] for age-incidence of clinical malaria	Fitted to age-incidence of clinical malaria for Dielmo and Ndiop in Senegal[23], and Idete, Tanzania[24]
Parameterization for severe disease and	Fitted to phase 3 trial for severity and overall mortality using case fatality rate (CFR) from the World Malaria Report[25]	Severe disease model[5] fitted to data from northern Tanzania[26] and to severe disease vs. prevalence relationship from data of multiple sites[27]. Mortality due to malaria is	Age incidence of severe malaria fitted to 5 sites in The Gambia and Kenya[29]. Proportion of severe disease from anemia and cerebral	Severe disease and mortality model5[16] fitted to all-cause and cause-specific age-specific mortality from pre-LLIN and pre-ACT era, to hospitalisation rates

mortality incidence		based on Africa-wide data from verbal autopsy and parasite prevalence[28].	malaria[30] CFR was normalized to match WHO mortality estimates.	by prevalence for multiple sites[27] and to age incidence of hospitalized severe malaria[29] (with age-specific CFR based on Tanzanian data32[26])
Vaccines intervention s	Pre-erythrocytic vaccines	Transmission blocking, pre-erythrocytic and combinations	Transmission blocking vaccines, blood stage vaccines, pre-erythrocytic and combinations	Transmission blocking vaccines, blood stage vaccines, pre-erythrocytic and combinations
Pre-erythrocytic vaccine effect in the model	Proportionate reduction in force of infection, exponential and bi-	Proportionate reduction in force of infection. Vaccine efficacy decays using exponential or biphasic decay, or using an antibody-based function informed by Phase II/III studies.	Proportionate reduction in force of infection, exponential decay.	Proportionate reduction in force of infection assuming beta distributed variation in efficacy. Various different assumptions concerning vaccine efficacy and its decay have been modeled, including exponential and biphasic-like (implemented via Weibull decay function)
Vector control Intervention s	Assumes that parasite prevalence levels represents prevalence under current levels vector control interventions without modelling them explicitly	LLIN, IRS, Larval control (larviciding & pupaciding) Novel interventions - GM mosquitoes, Ivermectin, Attractive Toxic Sugar Baits	LLIN, IRS, Larviciding-- effect depends on vector species-specific feeding behaviours Novel vector-control interventions: ivermectin, GM mosquitoes, individual and spatial repellents, oviposition traps, sugar-baited traps, etc.	LLIN, IRS, Larviciding, repellents and screening, zooprophylaxis, odour-baited traps, sugar-baited traps -- effect depends on vector species-specific feeding behaviors. Model includes loss of insecticide and development of holes in LLINs.
Treatment intervention s	Treatment of clinical disease and severe disease.	Treatment of clinical disease and severe disease, by specified drug and diagnostic.	Mass screen and treat, IPTi, IPTc/SMC and IPTp/IST for separate pregnancy model	Drugs (routine access, mass administration, age- and risk-group targeting, diagnostic-guided administration)
Treatment of clinical disease and	Mass screen and treat, MDA, IPTi, IPTc/SMC. Model allows for drug resistance.			

severe disease, by specified drug, facility level and diagnostic.				
Spatial dynamic model	No	Capacity to run full spatial model, including spatial interactions. For this exercise model was run independently at ADMIN1 level and aggregated, thus capturing spatial heterogeneity but not spatial interactions.	Yes	No
Predictions for country or geographic area	Yes	Yes (see above)	Yes	Yes. Based on MAP[31] prevalence, population and access to effective treatment by geographic area[32]
Super-infections	No	New infection takes priority over existing infection	Superinfections with each infection having its own antigenic repertoire (possibly partially overlapping)	Superinfections occur with summed parasite densities
Exposure	Age dependent exposure, restricted to 0-10y. Susceptibility to infection increases with age	Exposure varies both by age and between individuals.	Configurable age-dependent exposure functions.	Age dependent exposure with non-linear function describing relationship between exposure and infection, with exposure rate varying
Infection-blocking immunity	Infection-blocking immunity and immunity against severe malaria develop with the number of previous infections	Infection blocking immunity develops with exposure and age.	Minimal natural pre-erythrocytic immunity is attained through sustained exposure to infectious bites.	Infection-blocking immunity occurs only in those with very high cumulative exposure[14]
Blood stage infections	Blood stage immunity is acquired through exposure to	Blood stage immunity develops with exposure and age, reducing both	Blood-stage immunity is acquired through the	Blood stage immunity develops with cumulative exposure to

and immunity	blood stage infections, increasing with number of infections. Immunity acts against clinical and severe disease, with immunity to severe acquired faster than clinical disease.	detectability of infection and onwards infectiousness.	cumulative exposure to different malaria infections with varying but partly overlapping antigenic repertoires	parasite densities and malaria infections.
Duration of infection	Fixed duration of infection	Duration of infection is "Erlang-like" distribution (convolution of exponential distributions)	Duration of infection driven by strength of hyper-immune response to discrete repertoire of antigens presented by each clonal infection..	Duration of infection is log normal
Clinical disease and history of exposure	Immunity against clinical disease increases based on the number of previous infections (calibrated)	3 levels of risks of infection considered based on parasite prevalence[33]	A proportion of infected individuals go on to develop clinical disease. Immunity to clinical disease develops with exposure and age, and also has a maternally acquired component.	Clinical disease is triggered by pro-inflammatory cytokines in response to parasite density passing through a configurable pyrogenic threshold, down-regulated to specific antibody production.
Decay of natural immunity	No decay of naturally acquired immunity (calibrated)	Exponential decay of naturally acquired immunity	Capacity for antibody production to specific parasite antigens decays to memory levels upon clearing an infection	Original model included no decay of natural immunity. Three model variants in the ensemble include different functional forms for decay of immunity[18].
Infectiousness and gametocyte models	Human infectiousness not included, hence no change in transmission following intervention	Human infectiousness to mosquitos is a weighted sum over the different human infectious states. A time lag between asexual parasitemia and infectious gametocytemia accounts for the lag in gametocyte development.	Probability of infecting mosquito is sigmoidal function of gametocyte density. Inflammatory immune response limits infectiousness of individuals	Infectiousness depends on lagged asexual parasite densities and on presence of gametocytes in blood meal which is a stochastic function of gametocyte density. Both male and female gametocytes must be

				present to infect mosquitoes[20].

Table S 2. Key results of selected modelling studies of likely impacts of RTS,S

Primary output metric		Ref-erence	Vaccine properties, deployment modality, coverage	Setting	Methodology	Contribution	Point estimates	Key insights
Vaccine properties		[34]	- PEV<br>- children<br>- Low and high VE and duration of protection at constant overall vaccine efficacy<br>- Schedules including 3 and 4 doses; 4th dose at 26 and varied from 16 to 30 months<br>- EPI delivery<br>- Coverage for doses 1-3 80%, 20% drop-off from for 4th dose; varied from 50 to 90% in 5% increments	PfPR2-10 from 5 to 50%	-Imperial College IBM<br>- Generic transmission settings, perennial pattern<br>- Treatment coverage for clinical disease 40%	- Effects of vaccine properties on vaccine impact<br>- Comparison of current implementation of RTS,S/AS01 to fractional dose implementation	- At PfPR2-10 5%cumulative clinical events averted per 1000 people aged 0 to 20 is 33 for low initial efficacy and high duration of protection, 37 for current RTS,S implementation, and 42 for high initial efficacy and low duration of protection<br>- At PfPR2-10 30% the cumulative impact is estimated at 179, 220, and 248 cases averted	- Higher VE is more important than duration of protection<br>- At longer duration of protection vaccine benefit is shifted toward older age groups<br>- Greater impact with fractional dose RTS,S compared to current implementation especially with larger interval between 3rd and 4th doses<br>- Efficacy profiles most sensitive for clinical outcomes

		[35]	- PEV<br>- infants and children<br>- 3 and 4 dose schedules<br>- EPI delivery<br>- DTP3 coverage proxy<br>-20% drop-off between 3rd and 4th dose	43 SSA countries	- Vaccine properties for the primary schedule ascertained by MCMC fitting to phase 3 trial data<br>- 4th dose VE assumed to be the same as following 3rd dose<br>OpenMalaria<br>- MAP 2010 (Ref)<br>- Country patterns in health seeking and effectiveness of malaria case management<br>- Impact estimates representative of country epidemiological and health systems contexts<br>- Incremental to case management and control interventions<br>- 5, 10, 15 years	- Estimates of RTS,S/AS01 VE, decay shape, and HL from 18 months of follow-up from phase 3 trial<br>- Impact of RTS,S on malaria outcomes in 43 countries	- In children VE of 79.2% and HL with exponential decay of 1.12 years<br>- Administered in children in a 4 dose schedule the vaccine will avert 310 to 620 malaria deaths and 67,190 to 116,890 clinical episodes per 100,000 FV following 10 years	- Vaccine efficacy against clinical malaria declines faster than the efficacy against infection<br>- Decay in efficacy is the key parameter contributing the most uncertainty to impact predictions<br>- Vaccine impact is increasing and effectiveness is decreasing with transmission intensity<br>- Vaccine impact is decreasing and effectiveness increasing with effective treatment<br>- The distribution of transmission intensity within country is the main driver of impact<br>- In high transmission demographic projections, future malaria control, and vaccination coverage are key drivers of uncertainty
		[36]	- PEV<br>- infants and children<br>- 3 and 4 dose schedules	11 African RTS,S trial sites	- Trial cohorts simulated using OpenMalaria model assuming different vaccine profiles, health systems, and transmission settings<br>- Vaccine properties determined by Bayesian fitting of model predictions to site and	Time-course of vaccine protection in infants and children based on phase 3 data from 32 months of follow-up	- In infants VE against infection after 3 doses is 65%, exponential decay, HL 7.2 months<br>- In children VE against infection is 91%, Weibull decay, HL of 7.32 months	- Decay in efficacy against clinical disease is more rapid than that against infection because of age-shifts<br>- The decay in efficacy against clinical malaria is due to both decay of immunity for protection against sporozoites and acquisition of natural immunity against clinical disease in the control cohort

					time-specific incidence of clinical malaria<br>- ITT analysis of phase 3, 32 months follow-up or longer<br>- Incremental to high usage of ITNs and access to quality malaria case management		- Boosting dose returns protection to VE of 49-55% in both cohorts	- In the cohort efficacy against infection is close to zero 3 years after the start of the trial<br>- Lower clinical efficacy and more age-shifting in higher transmission<br>- Overall sustained benefit of vaccination of up to 4 years following the primary schedule
		[37]	- PEV<br>- VE 52%<br>- children<br>- 3 dose schedule<br>- RTS,S trial	Manhiça, Mozambique	OpenMalaria<br>- Vaccine properties for the primary schedule ascertained by fitting to Phase 2b trial data OpenMalaria<br>- Country seasonality pattern<br>- 4 cohorts, ages 1 to 4, 5000 children<br>- 6 moths	- Predictions of trial outcomes using stochastic model		- Vaccine acts by blocking a certain fraction of infections that would otherwise reach the erythrocytic stages<br>- Lower efficacy against clinical disease than infection<br>- Loss of acquired immunity due to reduction in FOI in vaccinated cohort<br>- Higher efficacy against severe disease than clinical disease<br>- VE is age dependent<br>- VE efficacy against severe episodes highest in smallest children with least acquired immunity<br>- Measurements of VE in trial is sensitive to seasonality pattern<br>- Interplay between vaccine-induced and naturally acquired immunity explains variations in efficacy between outcomes and trial sites<br>- Stochastic models capture well malaria vaccine dynamics and are appropriate to use for prediction of

								the likely impact of vaccination in across the transmission continuum
		[18]	- PEV<br>- VE 60%, HL 1,2 ,5, 10, 100 years<br>- 3 dose schedule<br>- infants<br>- EPI, EPI plus infant catch-up, EPI plus school-based boosting (one time), mass campaign (every 5 years),<br>- EPI coverage 89%, 50% and 80% all other strategies	EIR 2, 11, and 20 with seasonality pattern of Namawala, Tanzania	OpenMalaria<br>- Low effective coverage for uncomplicated malaria<br>- 10 years	Vaccine properties by fitting to trial data under varying assumptions on decay of immunity and heterogeneity in transmission [19]<br>- HL<br>- Partial efficacy of incomplete schedule	Various	- Clinical efficacy in the trial lower that the underlying VE due to age shifting<br>- Lower clinical efficacy under higher transmission heterogeneity<br>- Clinical efficacy profile characterized by an initial increase and then a decline in the first year of life consistent with maternal immunity<br>- Fitting a model ensemble yielded similar parameters related to severe morbidity and mortality<br>- Impact not sensitive to HL longer than 5 years<br>- For EPI modalities impact not sensitive on assumptions on partial efficacy of the incomplete schedule as coverage is high, important implications for mass vaccination
Impact		[38]	- PEV<br>- VE 50%, duration of immunity between 1 and 10 years<br>- all ages<br>- annual campaigns, 80% coverage	Pre-intervention P*f*PR varied from 20% to 98%	- Vaccine impact evaluated using a compartmental malaria transmission [39]	- Impact on elimination of infection when deployed singly or with ITNs, MSAT (ACT), MDA (PQ, TQ)<br>- Impacts evaluated in the presents	- At PfPR2-10 between 20% and 50% elimination is reached with vaccination deployed singly<br>- At PfPR2-10 between 50% and 75% elimination is reached with vaccination and	- Combining interventions can protect drugs from selective pressure through indirect effects on transmission dynamics<br>- For vaccine only strategies excess morbidity if elimination is not reached<br>- For vaccine or ITN and MSAT strategies at coverages above required for elimination will have a positive impact on selection pressure,

						and evolution of drug resistance	one other intervention<br>- At PfPR2-10 between 75% and 85% elimination is reached with vaccination and two other interventions	prevent or reverse spread of drug resistance<br>- Cessation of above strategies prior to reaching elimination will accelerate drug resistance<br>- Cessation of elimination strategy in low transmission in the absence of resistance will reduce clinical infection<br>- Cessation of elimination strategy in high transmission will result in rebound in clinical infection in the presence of drug resistance

		[40]	- PEV<br>- infants, school age children, all ages<br>- 3 and 4 dose schedules<br>- VE against infection 60%<br>- Partial VE of 50% and 40% after 2 and 1 dose respectively<br>- HL 10 years<br>- EPI, EPI plus catch-up (up to 18 months), EPI plus school (6 to 11 years), EPI plus mass campaign delivery<br>- coverage at 3rd dose 89% for EPI, 51% for all other deliveries	Seasonal malaria transmission with EIR between 2 and 20 and 7 patterns for transmission trends	OpenMalaria<br>- Transmission trends induced by changing IRS coverage<br>- 10 years	Effects of malaria transmission trends on vaccine impact	- Limited impact of vaccine on transmission<br>- EPI impact on cases and deaths not very sensitive to transmission trends<br>- Mass campaign impact most sensitive to transmission trends; highest in lowest or suppressed EIR	- Varying transmission trend results in shifts in age-pattern of the disease<br>- Impact changes almost linearly with number of doses administered<br>- Supplementing EPI with catch-up campaign most efficient in decreasing transmission<br>- Mass campaigns most efficient in low transmission; at EIR above 2 mass campaigns less efficient than EPI<br>- Relative importance of transmission trends is contingent on HL assumptions
		[3]	- PEV<br>- infants and all ages<br>- VE against infection 50%<br>- HL 3 years	6 settings with varying EIR	Imperial College IBM	Incremental impact of a malaria vaccine on reduction in parasite prevalence	- Marginal incremental impact on prevalence when added via EPI across settings	- If deployed in combinations current interventions can result in substantial declines in malaria prevalence across a wide range of transmission settings<br>- Prevalence below 1% can be reached with scale-up of LLINs and ACTs in low transmission, and LLIN,

			- EPI and mass campaign every 3 years<br>- 90% coverage	(5 to 500), seasonality, and species composition		when added singly or as part of an intervention package	and intervention packages<br>- Can reduce prevalence to below 1% in low transmission settings when added via mass campaign on top of LLIN scale-up or MSAT<br>- In moderate transmission substantial reduction in prevalence to below 5% when added on top of LLIN, IRS and MSAT scale-up<br>- In high transmission mass vaccination results in modest reduction in prevalence	ACTs, and IRS in medium; MSAT can speed-up prevalence reduction<br>- In high transmission current tools alone are not sufficient to reach pre-elimination<br>- Impacts are dependent on bionomics of local vectors, patterns of coverage and adherence
		[18]	- PEV<br>- VE 60%<br>- 3 dose schedule<br>- infants<br>- EPI, EPI plus infant catch-up, EPI plus school-	EIR 2, 11, and 20 with seas	OpenMalaria<br>- Low effective coverage for uncomplicated malaria<br>- 14 years	- Impact of a malaria vaccine in very low to moderate transmission settings		- EPI modalities minimal effect on transmission, elimination was not achieved with either strategy<br>- EPI modalities little effect on overall prevalence, some reduction in youngest age groups, mass

			based boosting (one time), mass campaign (every 5 years),<br>- EPI coverage 89%, 50% and 80% all other strategies	onal ity patt ern of Nam awal a, Tanz ania		-Deployment strategy most efficient for the transmission range		vaccination eliminated infection at lowest EIR<br>- EPI modalities averted a modest number of malaria episodes; these effects are robust to assumptions on immunity and heterogeneity in transmission. Substantial reductions in morbidity with mass vaccinations with decreasing returns over time<br>- EPI modalities only modest effects on severe outcomes, large effects for mass campaign with decreasing returns over time<br>- Uncertainty in impact estimates increases over time<br>- EPI the most efficient strategy (highest impact per 1000 doses) in EIR 20, and 11, at EIR 2 mass vaccination is highest impact
		[41]	- PEV<br>- infants<br>- 3 dose schedule<br>- VE against infection 63%, HL 1.12 years, exponential decay<br>- EPI delivery<br>- 93% coverage at 3rd dose	EIR valu es (8) fro m 0.1 to 256 with a seas onal	OpenMalaria<br>- Effective coverage for uncomplicated malaria 14.5%<br>- Transmission held constant<br>- 20 years	- Age and magnitude of age- and time-shifting of incidence<br>- Effects of transmission intensity on age and magnitude of age- and time-shifting effects	- Excess incidence of uncomplicated disease in all age groups between 5 and 20 years of age<br>- The first observed onset of excess episodes for 5-6 year old age group occurs as early as 4-5 years after the	- The overall impact of the program on disease burden is positive<br>- At very low EIR the first onset of excess uncomplicated disease can be as late 10 years after the start of the program<br>- The large bulk of vaccine impact is concentrated in the 0-4 age group<br>- Maintaining the program at a constant level for an extended period of time leads to a new steady state with an age-shift in the pattern of the disease

				transmission pattern			start of the vaccination<br>- First onset of excess cases happens sooner at higher EIR	- Population immunity decays slowly; age-shifts result from recruitment of unexposed infants<br>- Increase in force of infection with age magnifies age-shifting effects<br>- Timing and extent of age-shifts is sensitive to birth-rate and age distribution
		[41]	- PEV<br>- infants<br>- 3 dose schedule<br>- VE against infection 63%, HL 1.12 years, exponential decay<br>- EPI delivery<br>- 93% coverage at 3rd dose	EIR values (8) from 0.1 to 256 with a seasonal transmission pattern	OpenMalaria<br>- Effective coverage for uncomplicated malaria 14.5%<br>- Transmission held constant<br>- 20 years	- Age and magnitude of age- and time-shifting of incidence<br>- Effects of transmission intensity on age and magnitude of age- and time-shifting effects	- Excess incidence of uncomplicated disease in all age groups between 5 and 20 years of age<br>- The first observed onset of excess episodes for 5-6 year old age group occurs as early as 4-5 years after the start of the vaccination<br>- First onset of excess cases happens sooner at higher EIR	- The overall impact of the program on disease burden is positive<br>- At very low EIR the first onset of excess uncomplicated disease can be as late 10 years after the start of the program<br>- The large bulk of vaccine impact is concentrated in the 0-4 age group<br>- Maintaining the program at a constant level for an extended period of time leads to a new steady state with an age-shift in the pattern of the disease<br>- Population immunity decays slowly; age-shifts result from recruitment of unexposed infants<br>- Increase in force of infection with age magnifies age-shifting effects<br>- Timing and extent of age-shifts is sensitive to birth-rate and age distribution
		[10]	-PEV, TBV<br>- range of VE, HL 4 years,	3 settings with	EMOD IDK<br>- Epidemological setting described by Namawala, Tanzania	Impact on transmission and prevalence	- PEV at 90% VE reduces both EIR and prevalence only at very low	- Maximum-impact of vaccines where vector control is saturated

			exponential decay<br>- all ages<br>- mass campaign, bi-annual<br>-90% coverage	varying EIR and seasonality pattern	- IRS killing efficacy 0.8, HL 1 year, annual campaign, 70% coverage<br>- Ignores case management<br>- 10 years	when deployed singly and with IRS	transmission, minor perturbations otherwise<br>- Reductions in prevalence of up to <10% when high efficacy PEV or TBV are deployed along with IRS	- Vaccines are most impactful when deployed at low transmission or combined with vector control<br>- Temporal dynamics of intervention efficacy are essential for deciding on frequency of distribution and timing of rebound due to efficacy decay<br>- Vaccine impact is modified in important ways by mixture of vector species, their ecologies, and behaviors both at baseline and over time
		[11]	-PEV, TBV<br>- 50% and 90% VE, HL 5 years, constant decay<br>- all ages<br>- mass campaign, one time<br>- EPI, one time mass vaccination of under-5 years old, thereafter newborns only<br>- 50% and 80% coverage	EIR range from 0 to 150, seasonal	EMOD IDK<br>- Epidemological setting described by Namawala, Tanzania<br>- ITN 0.9 blocking indoor feeds, 0.6 killing efficacy, continuous distribution, 80% coverage<br>- Independent distribution of interventions<br>- Ignores case management<br>- 10 years	- Impact on inoculation rates, prevalence, clinical outcomes and interruption of transmission when deployed singly and with ITN<br>- Settings where vaccine impact is maximized	- High potential impact for PEV deployed singly via mass campaign at EIR 15 and with 50% indoor feeding<br>ADD more results???	- Maximum-impact of vaccines where ITN coverage is saturated, primarily outdoor feeding vectors, low transmission<br>- Where ITN coverage is low or moderate and vectors are predominately indoor-biting scaling up ITN before vaccine introduction will increase effectiveness of vaccination<br>- Heterogeneity in biting exposure lowers impact of vaccination
Cost		[42]	- PEV<br>- children	Tanzania	- Cost inclusive of commodities, storage and	Estimate of cost of	Cost per FIC from \$4.2 to \$31.2	- Vaccine price is main cost driver of the program

			- 3 dose schedule<br>- EPI delivery<br>- coverage equal to DTP3		distribution, planning, training, social mobilization, delivery<br>- Micro-costing methodology<br>- Populated with data from Tanzania immunization program<br>-2004, USD	introduction of a hypothetical malaria vaccine in an endemic country	when vaccine price is varied from $1 to $10 per dose	- At vaccine price of $6 per dose non-vaccine costs account for less than 10% of total economic program costs
		[43]	- PEV<br>-infants and children<br>- 3 and 4 dose schedules<br>- EPI delivery<br>- coverage equal to 75% of country DTP3, 20% drop-off between 3rd and 4th dose	Burkina Faso Ghana Kenya Senegal Tanzania Uganda	- Cost inclusive of commodities, storage and distribution, planning, training, social mobilization, delivery<br>- Micro-costing methodology<br>- Populated with data from country cMYP, WHO-CHOICE<br>- 2013, USD	- Estimates of cost of *RTS,S* malaria vaccine introduction in 6 endemic countries<br>- Impact of country contexts on program costs	Cost per FIC (3-dose schedule) from $23.1 to $28.3 at assumed vaccine price of $5 per dose	- Vaccine price is main cost driver of the program<br>- At vaccine price of $5 per dose non-vaccine costs account for 5% of total financial and 16% of economic program costs<br>- Differences in program costs between countries driven by coverage, structure of the EPI program, and cost of labor
Cost-effectiveness		[44]	- PEV<br>- infants<br>- 3 dose<br>- VE 52%<br>- HL 10 years<br>- EPI delivery<br>- 89% coverage for doses 1-2, 6% drop-off for dose 3	Tanzania	OpenMalaria<br>- Impact estimates representative of country epidemiological and health systems contexts<br>- Impact and costs incremental to case management<br>- Case management of uncomplicated malaria 5%	- Estimate of ICER of a hypothetical malaria vaccine in an endemic country<br>- Impact on ICER of different	ICER ranges from $12 to $120 per DALY averted when vaccine price is varied from $1 to $20 per dose	- ICER changes linearly with vaccine price<br>- ICER increases over time<br>- ICER is lower at higher transmission intensities<br>- ICER changes linearly with VE<br>- ICER is similar for vaccine profiles with a HL of 6 months and one year<br>- ICER is halved for vaccine profiles with a HL of two years

					- Country program costs from[42]<br>- Country cost of case management from [45]<br>- Societal perspective<br>- 20 years<br>- 2004, USD	vaccine properties<br>- Vaccine impact on anemia<br>- Vaccine impact on productivity gains		- Decreasing returns on extending HL beyond two years<br>- ICER is lower when efficacy is concentrated in fewer individuals<br>- ICER changes linearly with coverage<br>- Savings resulting from productivity gains are negligible when vaccine price per dose is at or above $4
		[46]	- PEV, BSV, MSTBV and combination vaccines<br>- infants and infants and all ages<br>- schedules including 3 doses, 3 doses and boosters at 1, 2, 3, and 4 years<br>- EPI, and EPI and mass campaign<br>- VE varied from 0% to 100%<br>- HL 10 years<br>- 89% coverage for doses 1-2, 6% drop-off for dose 3	EIR values of 5.25, 21, 42, 168	OpenMalaria<br>- Incremental to case management<br>- Generic transmission settings, no seasonality<br>- Case management of uncomplicated malaria 5%<br>- Generic program costs from[42] adjusted for inflation<br>- Cost of case management from multiple sources (based on Tanzania) adjusted for inflation<br>- Societal perspective<br>- 10 years<br>- 2007, USD	- Impact of different vaccine types and their combinations<br>- Impact of different schedules<br>-Impact of different deployment modalities	ICER below $50 per DALY averted for a 3-dose PEV delivered to infants across EIRs and vaccine efficacies at assumed vaccine price of $2 per dose	- Adding boosters to EPI yields higher ICER for all vaccine types and across transmission settings<br>- Adding mass vaccination at moderate coverage to EPI results in lower ICER at lower EIR, and higher ICER in moderate to high EIR<br>- BSV is more effective and cost-effective than PEV in high EIR; the opposite holds for low EIR<br>- Combining PEV with BSV yields lower ICER<br>- EPI modalities of combination of MSTBV with either PEV or BSV or both do not yield additional effectiveness over a single vaccine type<br>- Mass campaigns of MSTBV with either PEV or BSV or both yield significant effectiveness gains and lower ICER over a single vaccine type

		[47]	- PEV<br>- infants<br>- 3 dose schedule<br>- VE 60%<br>- HL 10 years<br>- EPI delivery schedule<br>- 89% coverage for doses 1-2, 6% drop-off for dose 3	EIR values (10) ranging from 0.131 to 420	OpenMalaria<br>- Incremental to case management<br>- Epidemiological parameters sampled from log-normal distributions with means and SDs estimated from fitting<br>- Health systems inputs sampled distributions evaluated from the literature<br>- Societal perspective<br>- 10 years<br>- 2008, I$	- Uncertainty in and contribution to of epidemiological parameters, health systems inputs, and vaccine properties on ICER<br>- EVPI<br>- Overall ICER for vaccine implementation in SSA over distribution of EIR of in the population	- Weighted average ICER over 15 EIR of I$111 to 145 per DALY averted at assumed vaccine price of $5 per dose<br>- Including parameter uncertainty yielded weighted average ICER of I$158 (106-241) to 200 (131-314) per DALY averted	- Program costs substantially exceed treatment health savings<br>- ICER is lowest at EIR 10<br>- ICER is optimal at EIR between 2 and 20<br>- ICER is high at very low and very high transmission intensities<br>- Over half of parameterizations yielded were below a ceiling ratio of I$207, and all but one – below a ceiling ratio of I$2008<br>- Main contributors to ICER uncertainty are transmission intensity, price per vaccine dose, decay rate of the vaccine effect<br>- EVPI is substantial
		[22]	- PEV<br>- children<br>- 3 and 4 dose schedules<br>- EPI delivery<br>- 90% coverage for doses 1-3	PfPr2-10 from 3% to 65%	- Vaccine impact simulated using OpenMalaria, EMOD-DTK, Imperial, GSK models<br>- Generic transmission settings, no seasonality<br>- Case management of uncomplicated malaria 45%	Consensus impact modelling of *RTS,S* from 4 mathematical models of malaria epidemiology and control	Median (range) ICER across model medians for 3 and 4 dose schedules is $30 (18-211) and $25 (16-222) per DALY averted at assumed vaccine price of $5	- ICER below $100 at assumed vaccine price of $5 per dose at PfPr2-10 above 10%<br>- Substantially higher ICER at PfPr2-10 below 10%<br>- Similar ICER for 3 and 4 dose schedules<br>- Less consensus between models on estimated ICER at low PfPr2-10

			-20% drop-off between 3rd and 4th dose		- Coverage of effective case management of severe malaria of 48%<br>- Program costs and treatment costs include only cost of commodities<br>- Provider perspective<br>- 15 years<br>- 2015, USD		per dose at PfPr2-10 above 10%	
		[48]	- PEV<br>- children<br>- 4 dose schedule<br>- Vaccine properties based on 76 months follow-up from phase 3 trial<br>- EPI delivery<br>- DTP3 coverage proxy<br>-20% drop-off between 3rd and 4th dose	43 SSA countries	OpenMalaria<br>- Country patterns in health seeking and effectiveness of malaria case management<br>- Impact estimates representative of country epidemiological and health systems contexts<br>- Incremental to case management and control interventions<br>- Program costs based on median value from[43]<br>-Treatment health savings based on country cost of care<br>- Provider perspective<br>- 10 years<br>- 2015, USD	Effects of country epidemiological and health systems contexts on ICER	Median ICER of $136 (range $116–$220) per DALY averted at assumed vaccine price of $5 per dose for countries with PfPr2-10 above 10%	- Lowest ICER in settings with PfPr2-10 between 10 and 40%<br>- Health systems inputs vary greatly within narrow transmission ranges<br>- Health systems inputs have important implications for impact and ICER<br>- Treatment health savings are marginal
		[19]	- PEV<br>- children<br>- 3 dose schedule	Seasonal and non-	OpenMalaria<br>- Case management assumptions from [45]	- Impact of a malaria vaccine on transmission,	- Minimal impact on transmission<br>- Average effectiveness over	- No community effects<br>- Reduced incidence of malaria morbidity and mortality in under 5s that persist over 20-year life span

			- VE against infection 52%<br>- Partial VE of 46% and 40% after 2 and 1 dose respectively<br>- HL 10 years<br>- EPI<br>- coverage at 3rd dose 89% for EPI, 51% for all other deliveries	seasonal transmission with EIR of 21 (reference), 5.1, and 82	- Incremental to case management<br>- 5, 10, 20 years	age incidence of clinical outcomes<br>- Effects of vaccine properties on vaccine impact<br>-Effects of transmission intensity and seasonality on vaccine impact	20 year period in preventing uncomplicated episodes and deaths are 0.067 and 0.12 respectively<br>- Changes in efficacy and coverage translate to near linear changes in effectiveness for all outcomes<br>- Effectiveness against uncomplicated disease nearly proportional to HL<br>- Effectiveness against severe disease and mortality is highest at HL of 2 years, little further improvement in effectiveness from extending HL to 5 and 10<br>- Highest effectiveness in higher transmission	- Effectiveness of the program changes over time<br>- Excess morbidity and mortality due to delayed acquisition of immunity in higher prevalence settings<br>- Substantial public health benefits possible with a leaky vaccine

							although effectiveness decays over time<br>- Effectiveness against uncomplicated episodes is higher in seasonal settings; marginal impact on severe outcomes	
		[49]	- PEV, BSV, MSTBV and combination vaccines<br>- infants and infants and all ages<br>- schedules including 3 doses, 3 doses and boosters at 1, 2, 3, and 4 years<br>- EPI, and EPI and mass campaign<br>- VE varied from 0% to 100%<br>- HL 10 years<br>- 89% coverage for doses 1-2, 6% drop-off for dose 3	EIR values of 5.25, 21, 42, 168	OpenMalaria<br>- Incremental to case management<br>- Generic transmission settings, no seasonality<br>- Case management of uncomplicated malaria 5%<br>- 10 years	- Impact of a malaria vaccine on transmission, age incidence of clinical outcomes<br>- Impact of different vaccine types and their combinations<br>- Impact of different schedules<br>- Impact of different deployment modalities	- PEV with VE of 50% delivered via EPI averts 13% of malaria deaths, via EPI w/booster – 15%, and 21% via mass campaign if coverage is 70%<br>- PEV highest impact in lower transmission (EIR<10.5)<br>- BSV prevalence reduction is highest in high transmission; at lower EIR similar to PEV<br>- Combinations of PEV and BSV have marginally greater	- High efficacy vaccines of any type reduce transmission (down to elimination if delivered via mass campaign), low and medium efficacy vaccines don't<br>- Even a moderately efficacious vaccine delivered via EPI will have a substantial impact on the number of clinical events<br>- Rebound effects for all vaccine types in higher transmission; greater rebound if delivered via mass campaign<br>- Effectiveness of PEV, BSV delivered via EPI depends on duration of protection for vaccines with HL less than 2-3 years<br>- Vaccines avert more clinical episodes and deaths if response to the vaccine is heterogeneous

							impact than either singly<br>- Herd immunity can be achieved with either PEV or BSV delivered via mass campaign especially if combined with MSTBV<br>- If VE is low adding booster to EPI or delivery via a mass campaign yield only marginal additional impact; impact stronger if HL is short	

		[1]	- PEV<br>- infants and children<br>- 3 dose schedule<br>- VE against infection 37.6% and HL 6.2 years in infants<br>- VE against infection 58.2% and HL 14.4 years in children<br>- Efficacy after 2 doses 25% of full schedule<br>- EPI delivery<br>- DTP3 coverage for infants and 75% of DTP3 coverage for children	42 SSA cou ntrie s	- An individual-based Markov cohort model<br>- 2010 MAP<br>- Country case management access from DHS and other published sources<br>- Management of severe disease fixed at 54% for all countries<br>- Incremental to case management<br>- No seasonality<br>- 10 years		- In infants over 5 million clinical cases, 119000 severe cases, 98600 hospitalizations, and 31000 deaths averted<br>- In children over 12.5 million clinical cases, 250000 severe cases, 208000 hospitalizations, and 65400 deaths averted	- Greater reduction in incidence of clinical malaria in low transmission<br>- Most benefit to occurs in 1-4 year olds<br>- Somewhat higher incidence of clinical malaria after older ages (above 5) in high transmission<br>- Impact estimates most sensitive to assumptions on waning decay and case-fatality rate<br>- Impacts on severe outcomes including mortality are highly uncertain
Cost-effectiv eness		[50]	- PEV<br>- children<br>- 4 dose schedule<br>- Vaccine properties based on 76 months follow-up from phase 3 trial<br>- EPI delivery<br>- Coverage varied in 10%	- Stra ta defi ned by PfPR , seas onal ity, vect	- Imperial College IBM<br>- LLIN: 3 yearly distributions<br>- IRS: DDT-like, annual<br>- SMC: SP-AQ, 6months to 5 years, 3 doses per month, 2 rounds per year<br>- Intervention costs including treatment sourced from literature<br>- Assumed vaccine price $5 per dose	- The relative cost-effectiveness of introducing and scaling-up RTS,S compared with further scale-up of LLIN, IRS, SMC		-Across settings LLIN coverage is to be maxed out before other interventions are introduced<br>- Cost-effective to switch to other interventions at LLIN coverage of 55-65%<br>- At $5 per dose RTS,S is second only in very high and very low non-seasonal settings, and preceded by vector control and seasonal chemoprevention in all but very low seasonal settings

			increments from 0% to 80%<br>-20% drop-off between 3rd and 4th dose	or spec ies and bion omi cs	- Incremental to case management<br>- Compared to scale-up of LLIN, IRS, and SMC<br>- Provider perspective<br>- 10 years<br>- 2015, USD<br>- Scale-up from 0% to next level in 10% increment optimizing ICER over 4 interventions	- Costs take into account diminishing marginal returns to scale<br>- Correlated and independent distribution of interventions		- When allowing for diminishing returns to scale RTS,S is introduced earlier when coverage of LLIN and other control interventions is at moderate levels<br>- At $3 per dose RTS,S comparable ICER to IRS and SMC<br>- At $1 per dose RTS,S comparable ICER to LLIN in half of the settings

References

1. Sauboin CJ, Van Bellinghen LA, Van De Velde N, Van Vlaenderen I: **Potential public health impact of RTS,S malaria candidate vaccine in sub-Saharan Africa: a modelling study.** *Malaria Journal* 2015, **14**.
2. Sauboin C, Van Bellinghen L-A, Van De Velde N, Van Vlaenderen I: **Economic Impact of Introducing the RTS,S Malaria Vaccine: Cost-Effectiveness and Budget Impact Analysis in 41 Countries.** *MDM Policy & Practice* 2019, **4:**2381468319873324.
3. Griffin JT, Hollingsworth TD, Okell LC, Churcher TS, White M, Hinsley W, Bousema T, Drakeley CJ, Ferguson NM, Basanez MG, Ghani A: **Reducing Plasmodium falciparum malaria transmission in Africa: a model-based evaluation of intervention strategies.** *PLoS Med* 2010, **7**.
4. Griffin JT, Ferguson NM, Ghani AC: **Estimates of the changing age-burden of Plasmodium falciparum malaria disease in sub-Saharan Africa.** *Nature Communications* 2014, **5:**3136.
5. Griffin JT, Hollingsworth TD, Reyburn H, Drakeley CJ, Riley EM, Ghani AC: **Gradual acquisition of immunity to severe malaria with increasing exposure.** *Proc Biol Sci* 2015, **282**.
6. White MT, Bejon P, Olotu A, Griffin JT, Bojang K, Lusingu J, Salim N, Abdulla S, Otsyula N, Agnandji ST, et al: **A combined analysis of immunogenicity, antibody kinetics and vaccine efficacy from phase 2 trials of the RTS,S malaria vaccine.** *BMC Med* 2014, **12:**117.
7. Eckhoff PA: **A malaria transmission-directed model of mosquito life cycle and ecology.** *Malar J* 2011, **10:**303.
8. Eckhoff PA: **Malaria parasite diversity and transmission intensity affect development of parasitological immunity in a mathematical model.** *Malar J* 2012, **11:**419.
9. Eckhoff P: **P. falciparum infection durations and infectiousness are shaped by antigenic variation and innate and adaptive host immunity in a mathematical model.** *PLoS One* 2012, **7:**e44950.
10. Eckhoff P: **Mathematical Models of Within-Host and Transmission Dynamics to Determine Effects of Malaria Interventions in a Variety of Transmission Settings.** *American Journal of Tropical Medicine and Hygiene* 2013, **88:**817-827.
11. Wenger EA, Eckhoff PA: **A mathematical model of the impact of present and future malaria vaccines.** *Malaria Journal* 2013, **12**.
12. McCarthy KA, Wenger EA, Huynh GH, Eckhoff PA: **Calibration of an intrahost malaria model and parameter ensemble evaluation of a pre-erythrocytic vaccine.** *Malar J* 2015, **14:**6.
13. Smith T, Killeen GF, Maire N, Ross A, Molineaux L, Tediosi F, Hutton G, Utzinger J, Dietz K, Tanner M: **Mathematical modeling of the impact of malaria vaccines on the clinical epidemiology and natural history of plasmodium Falciparum malaria: Overview.** *American Journal of Tropical Medicine and Hygiene* 2006, **75:**1-10.
14. Smith T, Maire N, Dietz K, Killeen GF, Vounatsou P, Molineaux L, Tanner M: **Relationships between the entomological inoculation rate and the force of infection for *Plasmodium falciparum* malaria.** *Am J Trop Med Hyg* 2006, **75 (Suppl 2):**11-18.
15. Smith T, Ross A, Maire N, Rogier C, Trape JF, Molineaux L: **An epidemiologic model of the incidence of acute illness in Plasmodium falciparum malaria.** *American Journal of Tropical Medicine and Hygiene* 2006, **75:**56-62.
16. Ross A, Maire N, Molineaux L, Smith T: **An epidemiologic model of severe morbidity and mortality caused by Plasmodium falciparum.** *American Journal of Tropical Medicine and Hygiene* 2006, **75:**63-73.
17. Chitnis N, Hardy D, Smith T: **A Periodically-Forced Mathematical Model for the Seasonal Dynamics of Malaria in Mosquitoes.** *Bulletin of Mathematical Biology* 2012, **74:**1098-1124.
18. Smith T, Ross A, Maire N, Chitnis N, Studer A, Hardy D, Brooks A, Penny M, Tanner M: **Ensemble Modeling of the Likely Public Health Impact of a Pre- Erythrocytic Malaria Vaccine.** *Plos Medicine* 2012, **9**.

19. Maire N, Tediosi F, Ross A, Smith T: **Predictions of the epidemiologic impact of introducing a pre-erythrocytic vaccine into the expanded program on immunization in sub-Saharan Africa.** *Am J Trop Med Hyg* 2006, **75:**111-118.
20. Ross A, Killeen GF, Smith T: **Relationships of host infectivity to mosquitoes and asexual parasite density in *Plasmodium falciparum*.** *Am J Trop Med Hyg* 2006, **75 (Suppl 2):**32-37.
21. Ross A, Smith T: **The effect of malaria transmission intensity on neonatal mortality in endemic areas.** *American Journal of Tropical Medicine and Hygiene* 2006, **75:**74-81.
22. Penny MA, Verity R, Bever CA, Sauboin C, Galactionova K, Flasche S, White MT, Wenger EA, de Velde NV, Pemberton-Ross P, et al: **Public health impact and cost-effectiveness of the RTS,S/AS01 malaria vaccine: a systematic comparison of predictions from four mathematical models.** *Lancet* 2016, **387:**367-375.
23. Trape JF, Rogier C: **Combating malaria morbidity and mortality by reducing transmission.** *Parasitol Today* 1996, **12:**236-240.
24. Kitua A, Smith T, Alonso PL, Masanja H, Urassa H, Menendez C, Kimario J, Tanner M: **Plasmodium falciparum malaria in the first year of life in an area of intense and perennial transmission.** *Trop Med Int Health* 1996, **1:**475-484.
25. World Health Organisation: **World Malaria Report 2008.** 2008.
26. Reyburn H, Mbatia R, Drakeley C, Bruce J, Carneiro I, Oloni R, Cox J, Nkya WM, Lemnge M, Greenwood BM, Riley EM: **Association of transmission intensity and age with clinical manifestations and case-fatality of severe Plasmodium falciparum malaria.** *J Am Med Assoc* 2005, **293:**1461-1470.
27. Marsh K, Snow R: **Malaria transmission and morbidity.** *Parassitologia* 1999, **41:**241-246.
28. Rowe A, Rowe SY, Snow RW, Korenromp EL, Armstrong-Schellenberg JR, Stein C, Nahlen BL, Bryce J, Black RE, Steketee RW: **The burden of malaria mortality among African children in the year 2000.** *Int J Epidemiol* 2006.
29. Snow R, Omumbo J, Lowe B, Molyneux CS, Obiero JO, Palmer A, Weber MW, Pinder M, Nahlen B, Obonyo C, et al: **Relation between severe malaria morbidity in children and level of Plasmodium falciparum transmission in Africa [see comments].** *Lancet* 1997, **349:**1650-1654.
30. Okiro EA, Al-Taiar A, Reyburn H, Idro R, Berkley JA, Snow RW: **Age patterns of severe paediatric malaria and their relationship to Plasmodium falciparum transmission intensity.** *Malar J* 2009, **8:**4.
31. Bhatt S, Weiss DJ, Cameron E, Bisanzio D, Mappin B, Dalrymple U, Battle KE, Moyes CL, Henry A, Eckhoff PA, et al: **The effect of malaria control on Plasmodium falciparum in Africa between 2000 and 2015.** *Nature* 2015, **526:**207-+.
32. Galactionova K, Tediosi F, de Savigny D, Smith T, Tanner M: **Effective Coverage and Systems Effectiveness for Malaria Case Management in Sub-Saharan African Countries.** *Plos One* 2015, **10**.
33. Hay SI, Guerra CA, Gething PW, Patil AP, Tatem AJ, Noor AM, Kabaria CW, Manh BH, Elyazar IRF, Brooker S, et al: **A World Malaria Map: Plasmodium falciparum Endemicity in 2007.** *Plos Medicine* 2009, **6**.
34. Hogan AB, Winskill P, Verity R, Griffin JT, Ghani AC: **Modelling population-level impact to inform target product profiles for childhood malaria vaccines.** *Bmc Medicine* 2018, **16**.
35. Penny MA, Galactionova K, Tarantino M, Tanner M, Smith TA: **The public health impact of malaria vaccine RTS,S in malaria endemic Africa: country-specific predictions using Phase III data and simulation models.** *BMC Med* 2015, **13**.
36. Penny MA, Pemberton-Ross P, Smith TA: **The time-course of protection of the RTS,S vaccine against malaria infections and clinical disease.** *Malar J* 2015, **14**.
37. Maire N, Aponte JJ, Ross A, Thompson R, Alonso P, Utzinger J, Tanner M, Smith T: **Modeling a field trial of the RTS,S/AS02A malaria vaccine.** *American Journal of Tropical Medicine and Hygiene* 2006, **75:**104-110.

38. White LJ, Maude RJ, Pongtavornpinyo W, Saralamba S, Aguas R, Van Effelterre T, Day NPJ, White NJ: **The role of simple mathematical models in malaria elimination strategy design.** *Malaria Journal* 2009, **8**.
39. Ghani AC, Sutherland CJ, Riley EM, Drakeley CJ, Griffin JT, Gosling RD, Filipe JA: **Loss of population levels of immunity to malaria as a result of exposure-reducing interventions: consequences for interpretation of disease trends.** *PLoS One* 2009, **4:**e4383.
40. Brooks A, Briet OJT, Hardy D, Steketee R, Smith TA: **Simulated Impact of RTS,S/AS01 Vaccination Programs in the Context of Changing Malaria Transmission.** *Plos One* 2012, **7**.
41. Pemberton-Ross P, Smith TA, Hodel EM, Kay K, Penny MA: **Age-shifting in malaria incidence as a result of induced immunological deficit: a simmulation study.** *Malar J* 2015, **14**.
42. Hutton G, Tediosi F: **The costs of introducing a malaria vaccine through the Expanded Program on Immunization in Tanzania.** *American Journal of Tropical Medicine and Hygine* 2006, **75:**119-130.
43. Galactionova K, Bertram M, Lauer J, Tediosi F: **Costing RTS,S introduction in Burkina Faso, Ghana, Kenya, Senegal, Tanzania, and Uganda: A generalizable approach drawing on publicly available data.** *Vaccine* 2015, **33:**6710-6718.
44. Tediosi F, Hutton G, Maire N, Ross A, Tanner M: **Predicting the cost-effectiveness of introducing a pre-erythrocytic malaria vaccine into the expanded program on immunization in Tanzania.** *Am J Trop Med Hyg* 2006, **75:**131-143.
45. Tediosi F, Maire N, Smith T, Hutton G, Utzinger J, Ross A, Tanner M: **An approach to model the costs and effects of case management of Plasmodium falciparum malaria in sub-Saharan Africa.** *American Journal of Tropical Medicine and Hygiene* 2006, **75:**90-103.
46. Tediosi F, Maire N, Penny MA, Studer A, Smith TA: **Simulation of the cost-effectiveness of malaria vaccines.** *Malar J* 2009, **8:**127.
47. Maire N, Shillcutt SD, Walker DG, Tediosi F, Smith TA: **Cost-effectiveness of the Introduction of a Pre-Erythrocytic Malaria Vaccine into the Expanded Program on Immunization in Sub-Saharan Africa: Analysis of Uncertainties Using a Stochastic Individual-Based Simulation Model of *Plasmodium falciparum* Malaria.** *Value Health* 2011, **14:**1028-1038.
48. Galactionova K, Tediosi F, Camponovo F, Smith TA, Gething PW, Penny MA: **Country specific predictions of the cost-effectiveness of malaria vaccine RTS,S/AS01 in endemic Africa.** *Vaccine* 2017, **35:**53-60.
49. Penny MA, Maire N, Studer A, Schapira A, Smith TA: **What Should Vaccine Developers Ask? Simulation of the Effectiveness of Malaria Vaccines.** *Plos One* 2008, **3**.
50. Winskill P, Walker PGT, Griffin JT, Ghani AC: **Modelling the cost-effectiveness of introducing the RTS,S malaria vaccine relative to scaling up other malaria interventions in sub-Saharan Africa.** *Bmj Global Health* 2017, **2**.
